# Effects of using four baskets during simulated youth basketball games

**DOI:** 10.1371/journal.pone.0221773

**Published:** 2019-08-23

**Authors:** Nuno Mateus, Bruno Gonçalves, Anthony Weldon, Jaime Sampaio

**Affiliations:** 1 Research Centre in Sports Sciences, Health Sciences and Human Development, CIDESD, CreativeLab Research Community, University of Trás-os-Montes e Alto Douro, UTAD, Vila Real, Portugal; 2 The Technological and Higher Education Institute of Hong Kong (THEi), Hong Kong; University of Brasilia, BRAZIL

## Abstract

This study aimed to identify how playing basketball with two additional baskets influences the players’ technical, physiological, physical and especially, positional performance. Fourteen youth players performed eight 5vs.5 simulated basketball games, four with the two official baskets and four with two-extra official baskets, each one placed in the court restricted area. The variables collected were technical (field-goals made and missed, offensive and defensive rebounds, steals, passes, dribble-drive, give-and-go and ball possessions), physiological (heart rate monotony and sample entropy), workload (total distance covered and distance covered at different velocities) and positioning-related (distance to the nearest opponent, distance to the nearest teammate, stretch-index and distance between centroids). The results showed that the four-baskets games favoured the emergence of individual behaviours, increasing the game' physical demands and promoting a collective dispersion, which might impair team playing. Conversely, when playing with two-baskets, there was less distance between teammates. In conclusion, this study has clear implications for practice as it emphasizes that coaches can manipulate the number of baskets to modulate training workload and promote different individual and team behaviours.

## Introduction

Team sports are complex, dynamic and physically demanding activities, requiring players to adapt behaviours, manage disorder and respond to emergent situations of cooperation and opposition [1, 2]. Therefore, coaches are required to develop effective training environments to maximise learning opportunities. Over the last decade, new training methodologies like the constraints-led approach that favours the interaction between the player, task and surrounding environment have been explored. The manipulation of the competitive environment (e.g., court configuration, scoring rules, numerical imbalance) [3–5], provides increased training variability, which has been shown to enhance the effectiveness of practice, and improve players' adaptability to perturbations in the competitive environment [6–8].

Constrained training tasks seem to produce similar perceptual-motor skills as competitive events, which may support the improvement of technical skills and physical fitness, and promote players’ decision-making by highlighting important information from the environment, which in turn leads to better tactical knowledge [9, 10]. For example, manipulating the number of scoring targets is a frequently used constraint, particularly in soccer, to expand the players’ breadth of attention and perceived stimuli, with implications on technical, physiological and physical demands, and team behaviour [11, 12]. Recently, Travassos, Goncalves [12] examined how changing the number of targets influenced first division soccer players’ collective behaviour. The authors found that an increased number of targets promoted greater interpersonal and group distance between opponents, teams played more cohesively, and different playing patterns emerged. Similarly, Fenoglio [13] compared the effects of using two or four scoring targets on young soccer players’ performance parameters, which identified that more targets lead to a greater number of scoring attempts, less total passes, and did not influence any other individual actions (e.g. dribbles). It has also been suggested that manipulating the scoring target is a useful strategy to manage physiological workload during exercise [14], as more scoring targets increases the randomness of youth soccer players’ cardiovascular demands (demonstrated by an increased randomness in heart-rate), which may more accurately imitate the fluctuations seen in competition and, ultimately, improve performance in various environments [15].

Recently, researchers have also aimed to understand how players and teams' positional behaviours fluctuate within the game context, such as how players' offensive and defensive behaviours change relative to the distance from scoring targets [16, 17]. It was revealed that players tend to be more coordinated close to their defensive areas (i.e. exhibiting a higher collaboration and nearness), perhaps aiming to stop the opposing teams initiatives; on the other hand, increased behaviour variability was observed near scoring zones, which may be an attempt to deceive and confuse the opponents’ defensive alignment [16]. Additionally, findings from Headrick, Davids [18], revealed that soccer defenders coordinate movements and shape their behaviours according to the distance between the attackers and the goal; while, basketball research, which examined players’ space-time patterns, showed that opposite players dyadic systems attracted to and repelled from each other, with defensive players constantly trying to close the space between themselves and the attacker, while also maintaining symmetry with the opposing player and the basket [19].

Although basketball is a very strategic game [20], where teams set up and run multiple standardized offensive plays and sophisticated defences with constant tactical adjustments throughout the game, there is a lack of information about the manipulation of game constraints, especially with regard to positional performance [21]. Previous basketball studies have identified the influence of modifying equipment, court dimensions and playing rules on players’ technical actions, physiological responses, workload, and its importance to improve game skills [3, 22, 23]. Also, individual and collective behaviours should be taken into account, since they provide a more comprehensive understanding of performance, enabling to access players’ perception, knowledge and interpretation in different scenarios [24]. Furthermore, basketball is characterized by multiple scoring opportunities, which increases the game pace and consequently intermittent high-intensity demands [25], however, to the best of our knowledge, no previous studies have evaluated the outcomes of increasing the number of baskets and its influence on creating additional scoring opportunities.

Therefore, it is important to further investigate how game constraints such as manipulating the number of scoring targets affects players’ responses, number of scoring opportunities, and exploration of new learning environments. Thus, we hypothesized that changing the number of baskets may affect players’ technical skills, workload, positional behaviour and personal-coordination tendencies. By this reasoning, an increase in unpredictable behaviours may be observed in offense, while a decrease in occupied space on the court when in defence, as a security response for the increased number of baskets [12, 18, 26]. Considering the above-mentioned information, the purpose of the current study was to identify the effects of manipulating the number of baskets (i.e., two-baskets *vs*. four-baskets), on young basketballers’ technical, physiological, physical and particularly, positional performance.

## Materials and methods

### Participants

A total of fourteen under-16 male players (age, 14.0 ± 0.9 years old; weight, 54.0 ± 9.3 kg; height, 173.0 ± 10.5 cm) from a regional-level basketball team participated in this study. Criteria for inclusion were applied to ensure all players were involved in three training sessions (with, at least, 90 minutes’ duration) and one competitive game per week. The training sessions had the following structure of warm-up; basketball drills, focusing on the acquisition and improvement of technical and tactical skills; basketball small-sided games; and 5-on-5 basketball games. An informed and written consent was provided to the coaches, players, and their parents before the beginning of the study. None of the players reported any musculoskeletal, neurological, or orthopaedic injury that might impair their participation. The study protocol was approved and followed the guidelines stated by the Ethics Committee of the of University of Trás-os-Montes and Alto Douro, based ate Vila Real (Portugal) and conformed to the recommendations of the Declaration of Helsinki.

### Design

**Fig 1:**
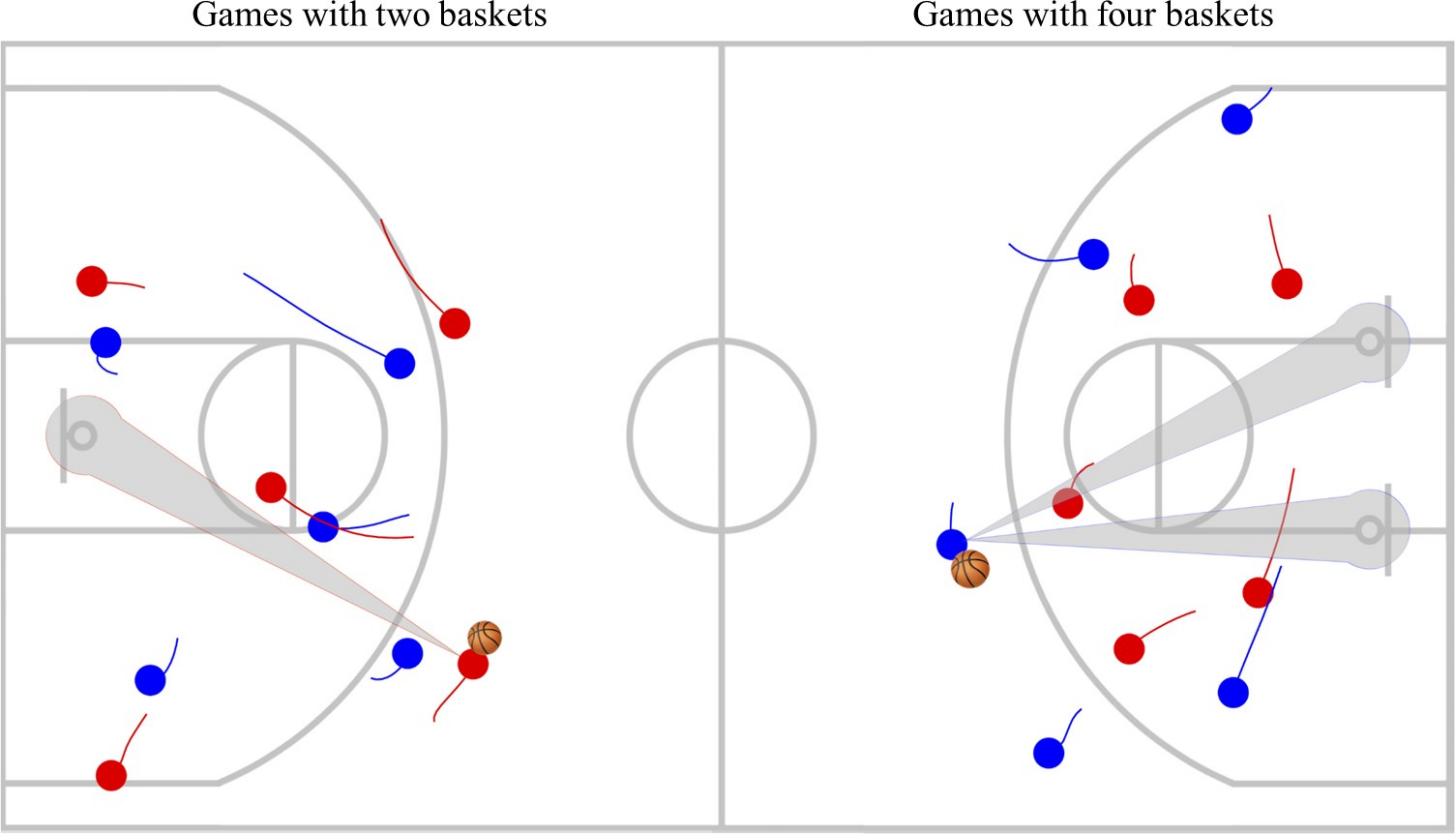
Representation of both game conditions with two official baskets and four official baskets, in a real-frame animation.

To ensure the assembly of balanced teams, the players were divided into four homogeneous teams, based on their skills, according to the coach’s perception about their passing ability, ball control, field-goal shooting and game knowledge. A total of eight 5*vs*.5 basketball games were performed during two preseason (September) training sessions in two different conditions: i) game with two official baskets and ii) game with four official baskets (see Fig 1). Each team participated in both game conditions per session, with all players participating in at least one game in each condition. The court characteristics were distributed arbitrarily per session, resulting in an overall of four games played in each condition. Every game was five minutes in duration, interspersed with a three-minute recovery period. The games were played at the beginning of a regular training session, after a fifteen-minute warm-up, consisting of submaximal running, dynamic stretching exercises and basketball lay-up drills. All players were previously familiarized with the two game situations. Both game conditions were refereed by the head coach and played in accordance with the official basketball rules. In each game, the players were instructed to attack according to their teams’ set plays, however, in defence, were asked to use half-court defence. To reduce the stoppage time, no free-throws were awarded and in the case of the ball going off, several balls were placed around the court to ensure its replacement was provided as fast as possible. Lastly, no feedback from coaches was allowed during the games.

### Data collection

All the game situations were video recorded using a digital camera (Sony CX625 Handycam®). Additionally, positional and heart-rate (HR) data of all players were collected using individual WIMU units (RealTrack Systems, Almería, Spain), with coupled heart-rate bands (Garmin, Soft Strap Premium, USA). Validity and reliability of WIMU® system have been reported previously and their operation and handling are documented elsewhere [27]. The mean absolute error of measurement is below 5.2 ± 3.1 cm for the x-position and 5.8 ± 2.3 cm for the y-position [27]. To decrease measurement error and increase the validity and reliability of the system, the players used the same unit across all the game situations.

### Data processing and derived-variables

The video files recorded with the digital camera were downloaded to a computer, and afterwards the following individual technical performance variables were registered: field-goals made (FGM), field-goals missed (FGMd), offensive rebounds (OREB), defensive rebounds (DREB) steals/interceptions (STL), passes (PASS), dribble drives (DD), give-and-go (G&G), and ball-possessions (BP). In order to ensure a high inter-rater reliability for all variables, the game analysis was inspected by two experienced basketball researchers and the results of inter-rater reliability were deemed as high (kappa coefficients >.90).

The physiological analysis consisted of using the players’ HR values for each game scenario in order to assess HR monotony and sample entropy (sampEn). Monotony, is commonly used as a measure of day-to-day training workload variation during a training week [28], was applied to measure the variation of players’ HR for each game. It was calculated by dividing the players’ average HR by the standard deviation of the HR over the game. On the other hand, sampEn was used to assess each players’ HR regularity during the games. SampEn (*m*, *r*, *n*) is defined as the negative natural logarithm of the conditional probability that two sequences, similar for *m* points (length of the vector to be compared), remain similar at the next point *m* + 1 [29]. The values used to calculate sampEn were 2 to vector length (*m*) and 0.2*SD to the tolerance (*r*) [30]. Values of sampEn range from zero towards infinity, where values close to zero were indicative of higher regularity in HR, while the higher the sampEn, the more unpredictable the HR.

The players’ spatial coordinates, collected by the WIMU units, were exported and computed using Matlab® software (MathWorks, Inc., Massachusetts, USA) [31]. The total distance covered, distance covered at different velocities and the game pace (i.e., mean speed for each player in each scenario) were measured as physical variables. The distance covered at different movement speeds were adapted from a previous basketball study [32] and standardized into the following four speed categories: walking (≤6 km/h); jogging (6.1–12 km/h); running (12.1–18 km/h); and sprinting (≥18.1 km/h). Additionally, the players’ load, number of total accelerations, total decelerations, and the number of high-intensity actions (number of jumps and impacts (> 5 G’s forces), accelerations (>2 m/s^2^) and decelerations (<-2 m/s^2^)) were calculated by the WIMU PRO system [33].

Furthermore, the positional data of players was also used to determine the following group and team positioning variables: distance to the nearest opponent (NearOP), distance to the nearest teammate (NearTM), stretch-index (SIX), and distance between centroids (DbC) [9, 34]. It should be noted that each of the variables was processed in order to calculate the average value and the coefficient of variation (CV), both for offense and defence phases.

### Statistical analysis

The differences between conditions of individual variables (i.e., technical, physiological and workload variables) were assessed using repeated samples parametric and non-parametric tests (t-test and Wilcoxon test). The collective variables (i.e., group and team behaviour variables) were processed with the corresponding independent tests (independent t-test and Mann-Whitney test). Statistical significance was set at p < .05 and calculations were carried out using SPSS software (IBM SPSS Statistics for Windows, Armonk, NY: IBM Corp.). Complementary, magnitude-based inferences and precision of estimation were applied. The individual differences were analysed with a specific repeated measures spreadsheet (post-only crossover trial) and the positional variables were compared using a spreadsheet for independent analysis (means of different groups’ comparison) [35]. All technical, physiological, workload and positional related variables effects were estimated in raw units and uncertainty in the estimate was expressed as 95% confidence limits. Smallest worthwhile differences were measured using the standardized units multiplied by 0.2 [36]. Uncertainty in the true effects of the conditions was evaluated with the non-clinical version of magnitude-based inferences. Probabilities were calculated qualitatively and described according to the following scale: >5%, unclear; 25–75%, possibly; 75–95%, likely; 95–99.5%, very likely; >99.5%, most likely. Standardized (*Cohen's d*) mean differences and respective 95% confidence intervals were also computed as magnitude of observed effects, and, thresholds were: 0–0.2, trivial; 0.2–0.6, small; 0.6–1.2, moderate; 1.2–2.0, large; and > 2.0, very large [37].

## Results

**Fig 2:**
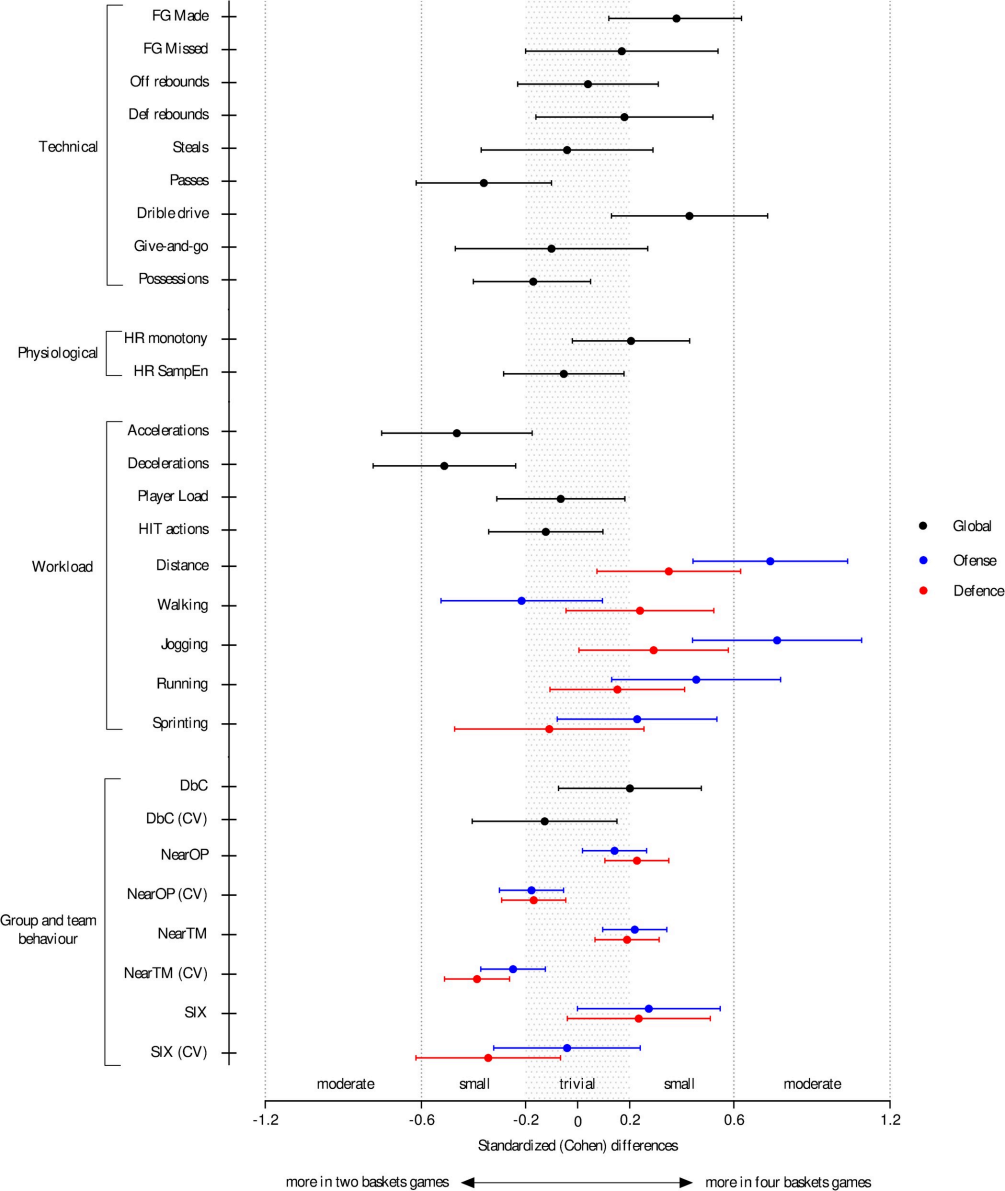
Standardized (Cohen) differences of technical, physiological, workload and group and team behaviour variables according to game condition analysis. Error bars indicate uncertainty in the true mean changes with 95% confidence intervals. Abbreviations: NearOP = distance to nearest opponent; NearTM = distance to nearest teammate; SIX = stretch-index; DbC = distance between centroids; CV = coefficient of variation.

Table 1 and Fig 2 present the comparison of outcomes for technical variables in both game scenarios. Actions such as FGM (0.4; ±0.3, Z = 34, p = 0.02, small effect; raw mean differences, ±95% CL) and DD (0.6; ±0.4, t = -2.4, p = 0.02, small effect) increased from the two-baskets game to the four-baskets game; on the other hand, the number of passes decreased (-1.1; ±0.8, t = 2.3, p = 0.03, small effect).

**Table 1:** Descriptive analysis of players' performance measures according to the number of baskets.

Variables	Condition (mean ± sd) (CV%)		Difference in means (%; ±95% CL)	Practical Inferences
	two-baskets	four-baskets		
**Technical**				
Field-goals made	0.47±0.9 (197.9)	0.87±1.2 (132.0)	0.4; ±0.3*	likely ↑
Field-goals missed	0.77±0.7 (96.5)	0.95±1.2 (127.8)	0.2; ±0.4	possible ↑
Offensive rebounds	0.33±0.6 (186.4)	0.36±0.6 (162.8)	0.0; ±0.2	unclear
Defensive rebounds	0.28±0.5 (180.9)	0.39±0.6 (153.4)	0.1; ±0.2	possible ↑
Steals	0.36±0.6 (162.8)	0.33±0.6 (173.2)	0.0; ±0.2	unclear
Passes	6.56±2.8 (42.5)	5.46±3.1 (57.1)	-1.1; ±0.8*	likely ↓
Dribble drive	0.85±1.0 (122.9)	1.41±1.4 (101.2)	0.6; ±0.4*	likely ↑
Give-and-go	0.05±0.3 (624.5)	0.03±0.2 (624.5)	0.0; ±0.1	unclear
Possessions	8.46±3.6 (42.8)	7.79±3.7 (47.9)	-0.7; ±0.9	possible↓

statistically significant differences at

*p < .05

**p < .01

***p < .001

The inferences of physiological and workload variables are shown in Table 2 and Fig 2. Regarding the physiological variables, between the two and four-baskets games, possible and likely-trivial differences were observed in HR monotony and HR sampEn, respectively. Whereas, the workload variables between the two and the four-baskets games shown a decrease in accelerations (-3.6; ±2.2, t = 2.7, p = 0.01, small effect) and decelerations (-3.9; ±2.1, t = 3.2, p = 0.003, small effect). Conversely the distance run in offense (10.6; ±4.3, t = -4.2, p = 0.001, moderate effect), defence (4.1; ±3.2, small effect), offensive jogging (5.4; ±2.3, t = -3.9, p = 0.001, moderate effect) and running (5.2; ±3.7, t = -2.4, p = 0.04, small effect), was higher in the four-baskets games.

**Table 2:** Descriptive analysis of players' performance measures according to the number of baskets.

Variables		Condition (mean ± sd) (CV%)		Difference in means (%; ±95% CL)	Practical Inferences
		two-baskets	four-baskets		
**Physiological**					
Heart-rate monotony		16.73±6.6 (42.8)	18.19±7 (38.5)	1.5; ±1.6	possibly ↑
Heart-rate sampEn		0.21±0.1 (43.6)	0.21±0.1 (32.5)	0.0; ±0	likely trivial
**Workload**					
Accelerations		85.64±7.9 (9.2)	82.05±6.9 (8.4)	-3.6; ±2.2*	likely ↓
Decelerations		85.51±8 (9.4)	81.62±6.5 (8.0)	-3.9; ±2.1**	very likely ↓
Player Load		7.77±1.3 (16.8)	7.68±1.2 (15.9)	-0.1; ±0.3	likely trivial
HIT actions		88.92±35.7 (40.2)	84.56±32.8 (38.8)	-4.4; ±7.8	possibly ↓
Distance covered	offense	97.80±15.2 (15.5)	108.42±12.1 (11.2)	10.6; ±4.3***	most likely ↑
	defence	94.19±10.8 (11.5)	98.28±11.5 (11.7)	4.1; ±3.2	likely ↑
Walking	offense	30.98±4.5 (14.5)	30.07±3.7 (12.2)	-0.9; ±1.3	possibly ↓
	defence	32.38±3.7 (11.3)	33.31±3.7 (11.2)	0.9; ±1.1	possibly ↑
Jogging	offense	37.32±7.2 (19.4)	42.69±6.1 (14.4)	5.4; ±2.3***	most likely ↑
	defence	38.48±8 (20.7)	40.86±7.6 (18.6)	2.4; ±2.3	possibly ↑
Running	offense	25.49±11.9 (46.5)	30.65±9.7 (31.7)	5.2; ±3.7*	likely ↑
	defence	19.42±6.8 (35.0)	20.59±7.8 (37.7)	1.2; ±2	possibly ↑
Sprinting	offense	5.23±4.5 (85.7)	6.43±5.6 (86.4)	1.2; ±1.6	possibly ↑
	defence	3.92±3.5 (90.4)	3.53±3.2 (91.8)	-0.4; ±1.3	unclear

statistically significant differences at

*p < .05

**p < .01

***p < .001

Results for the group and team behaviours are presented in Table 3 and Fig 2. Increasing the number of baskets possibly increased the NearOP defence (0.2; ±0.1, U = 55928, p = 0.004, small effect) and NearTM offense (0.3; ±0.2, U = 56274, p = 0.005, small effect); however possibly and very likely decreased the NearTM offense (CV) (-2.9; ±1.5, U = 55269, p = 0.002, small effect) and NearTM defence (CV) (-4.9; ±1.6, U = 51037, p = 0.001, small effect), respectively. Additionally, the four-baskets games were linked to a likely decrease of SIX defence (CV) (-3.2; ±2.6, t = 2.1, p = 0.04, small effect), but with a possible increase of offense and defence SIX (offense: 0.3; ±0.3, small effect; defence: 0.2; ±0.2, small effect) and DbC (0.2; ±0.3, small effect).

**Table 3:** Descriptive analysis of players' performance measures according to the number of baskets.

Variables			Condition (mean ± sd) (CV%)		Difference in means (%; ±95% CL)	Practical Inferences
			two-baskets	four-baskets		
**Group and team behaviour**						
Nearest Opponent	Average	offense	2.98±1.0 (34.8)	3.13±1.2 (37.9)	0.2; ±0.1	likely trivial
		defence	2.83±0.9 (31.1)	3.04±0.9 (32.8)	0.2; ±0.1**	possibly ↑
	CV	offense	43.98±15.5 (35.2)	41.27±15.2 (36.7)	-2.7; ±1.9*	possibly ↓
		defence	43.45±14.9 (34.4)	40.9±15.2 (37.2)	-2.6; ±1.9*	possibly ↓
Nearest Teammate	Average	offense	3.92±1.2 (30.7)	4.2±1.4 (32.2)	0.3; ±0.2**	possibly ↑
		defence	3.42±1.1 (31.8)	3.64±1.2 (33.7)	0.2; ±0.1*	possibly ↑
	CV	offense	35.78±12.2 (34.2)	32.86±11.3 (34.4)	-2.9; ±1.5**	possibly ↓
		defence	38.32±13.6 (35.4)	33.39±11.9 (35.8)	-4.9; ±1.6***	very likely ↓
Stretch Index	Average	offense	4.41±0.9 (19.5)	4.67±0.9 (20.7)	0.3; ±0.3	possibly ↑
		defence	3.87±0.8 (20.1)	4.07±0.9 (21.5)	0.2; ±0.2	possibly ↑
	CV	offense	20.38±8.2 (40.3)	20.07±6.9 (34.0)	-0.3; ±2.1	unclear
		defence	21.84±9.7 (44.3)	18.66±8.7 (46.7)	-3.2; ±2.6*	likely ↓
Distance btw centroids	Average		2.51±1.1 (41.8)	2.74±1.2 (44.0)	0.2; ±0.3	possibly ↑
	CV		47.49±19.5 (41.0)	45.14±17.4 (38.5)	-2.4; ±5.2	possibly ↓

statistically significant differences at

*p < .05

**p < .01

***p < .001

## Discussion

The current study aimed to assess how increasing the number of baskets (i.e., two-baskets *vs*. four-baskets) influences the technical, physical, physiological and especially, tactical profiles of players’, during 5*vs*.5 basketball games. The obtained results provided insightful information about how youth basketballers and teams performed according to the scoring target constraints. By adding extra baskets to the game influenced players’ individual performance, predominantly their technical actions, offense workload and positional behaviour, which consequently altered the teams’ playing patterns.

The primary outcome of this study was that informational scoring constraints are key to influencing players’ technical performance, reinforcing the importance of understanding the player-environment relationship. The higher number of baskets allowed more scoring opportunities, which subsequently increased field-goals made and dribble drives per player. Therefore, by applying such environments, coaches can provide learners with an array of information for perception and action, offering an optimal opportunity to improve their flexibility to adapt to environmental variations [8]. Moreover, especially in youth, settings that boost opportunities to score, promote perceptions of competence and enjoyment, increasing the players’ intrinsic motivation, a pivotal element to produce self-determined behaviour, effort, and persistence during the activity [38]. Conversely, the observed reduction in the passes performed can act as bias, particularly if coaches aim to enhance passing skills and off-ball actions essential to successfully receive a pass [3], as extra-baskets seem to favour the emergence of individual behaviours, with the players attempting to retain the ball, instead of employing a collective approach, as a result of more frequent and simple scoring opportunities.

Workload of training tasks is shaped by the environmental constraints imposed on the players’ cognitive process, whereby workload variables can fluctuate according to the interactions established between the performers and the surrounding environment [39]. The increase of scoring opportunities in the four-baskets games, lead to different physical demands, characterized by higher distances travelled and faster offensive displacements. Indeed, the increase of offense and defence SIX suggest that using additional baskets eventually promoted different playing patterns, with players positioning farther from each other, culminating in a higher workload. On the other hand, and in agreement with previous findings [32, 40], the traditional basketball game (i.e., two-baskets) required further lower-body explosive actions (i.e., accelerations and decelerations), perhaps as a result of a more organized pattern of play, demanding that players perform continuous accelerations and breaks (e.g., to start the dribble or a sudden movement of cut to the basket, to avoid defenders before shooting, to quickly react to offensive players’ actions), in order to gain advantage over opponents.

It is well known that fewer cooperative behaviours among the components of a biological system, can lead that system to new behavioural patterns [8]. Furthermore, higher game pace increases the number of activity responses, but impaired collective performance [41]. Additionally, the players positioning is a result of the perceived opportunities for action and the way they make use of the available information [42]. Within this context, the increase of both SIX and NearTM shown that the four-baskets games demanded a coadaptation of players’ offensive and defensive behaviours, influencing their positioning and consequently the teams' playing patterns. As aforementioned, it may be likely that the amplified exposure to scoring information favoured the emergence of individual behaviours, increasing the speed of offensive performance, consequently promoting team dispersion, thus impairing the team playing cohesively. Previous basketball research reported that larger distance between teammate's resulted in a less organized offense [43]. The decrease detected in NearTM (CV) and NearOP (CV) are in line with this reasoning, since higher variations in distances between players are usually a consequence of spontaneous interactions between attackers and defenders, occurring when players are more coupled, such as during custom basketball set plays [19].

The DbC observed reinforces this idea, revealing that teams played far away from each other. This event could be interpreted as a consequence of defensive players retreating to their position on the court to account for the disadvantage of defending two targets, and in an attempt to limit scoring opportunities by reducing the space surrounding the scoring targets [12]. However, in the current study, the increase of defensive NearTM proposed the opposite, emphasising that defensively teams did not behave more cohesively. In this sense, our results exposed that adding extra-baskets to a 5*vs*.5 basketball game, influenced the teams’ spatial-temporal relations, suggesting a reduction of organized and conservative set plays, in favour of more individualized, fast, dispersed and unbalanced playing patterns.

Although this study adds important findings regarding the influence of constraining training tasks on players’ movement behaviour, our exploratory results might represent the acute effects of the manipulation but were gathered with a small sample of a single team. Also, further research is needed to identify the long-term effects in learning and their transference to the formal game settings. One of the problems that may preclude coaching staffs from applying a similar approach in training is the lack of sufficient court baskets. A possible solution to promote a similar technical performance is to move both baskets to one half-court and start practising a half-court game with two-baskets. On the other hand, the use of vertical scoring areas during full-court games may be a suitable strategy to replicate identical physical demands and positional adaptations.

## Conclusions

Summarizing, this study presents new insights into the influence of manipulating scoring constraints on youth basketballers’ technical, physical and positional performance. The obtained results emphasized that the amplification of specific information during basketball games, can expand the players’ breadth of attention and perceived stimuli, facilitating individual technical performance. The subsequent adjustments promote an increase in physical demands and induce adaptations in players’ spatial-temporal relations, as well as the emergence of more dispersed and unbalanced behavioural patterns. In conclusion, this approach can be taken into account when designing training drills and modifying training periodization, especially to develop particular technical actions, increase workload and foster different team behaviours.
